# Care Seeking and Treatment of Febrile Children with and without Danger Signs of Severe Disease in Northern Uganda: Results from Three Household Surveys (2018–2020)

**DOI:** 10.4269/ajtmh.21-1132

**Published:** 2022-08-29

**Authors:** Phyllis Awor, Joseph Kimera, Nina C. Brunner, Proscovia Athieno, Gloria Tumukunde, Irene Angiro, Aita Signorell, Giulia Delvento, Tristan Lee, Maureen Amutuhaire, Jimmy Opigo, Flavia Mpanga Kaggwa, Fred Kagwire, Juliet Nakiganda, Christian Burri, Christian Lengeler, Manuel W. Hetzel

**Affiliations:** ^1^ Makerere University School of Public Health, Kampala, Uganda;; ^2^ Swiss Tropical and Public Health Institute, Allschwil, Switzerland;; ^3^ University of Basel, Basel, Switzerland;; ^4^ National Malaria Control Division, Ministry of Health, Kampala, Uganda;; ^5^ UNICEF Uganda Country Office, Kampala, Uganda;; ^6^ Clinton Health Access Initiative, Kampala, Uganda

## Abstract

Identification, stabilization, and prompt referral of children with signs of severe febrile disease (danger signs) in rural communities are crucial for preventing complications and death from severe malaria, pneumonia, and diarrhea. We set out to determine the treatment-seeking practices and treatment patterns for children < 5 years of age with an acute febrile illness, with or without danger signs of severe disease, in a highly malaria-endemic area of northern Uganda. Three household surveys were conducted from November through December each year in 2018, 2019, and 2020. Overall, 30% of the children in the study were reported to have had a WHO-classified danger sign including convulsions, unconsciousness/unusually sleepy, inability to feed or drink, and vomiting everything. Only half of the children in this study sought care from a health provider. However, significantly more children with danger signs of severe disease sought and received treatment and diagnostics from a health provider, compared with those without danger signs (adjusted odds ratio: 1.6, 95% confidence interval: 1.2–2.0; *P* < 0.01). In the total population studied, care seeking in the public sector was 26% and similar to care seeking in the private sector (24%). Community health workers were used as the first source of care by 12% of the children. Approximately 38% of the children who were reported to have danger signs of severe disease requiring prompt referral and treatment did not seek care from a health provider. Understanding and addressing barriers to accessing healthcare could contribute to better treatment seeking practices.

## INTRODUCTION

Malaria, pneumonia, and diarrhea together account for 50% of all deaths in children in the age group of 1 to 59 months, in sub-Saharan Africa.^1^^,^^2^ In Uganda, malaria is responsible for approximately 15% of deaths in children in this age group, pneumonia for another 15% and diarrhea for 10%.^3^ In addition, the prevalence of malaria, pneumonia, and diarrhea in children under 5 years admitted at health facilities in Uganda is 43%, 11%, and 5%, respectively.^4^ These three illnesses often present as an acute febrile illness with overlapping symptoms, and coinfection can occur.^5^^–^^7^

If untreated, malaria, pneumonia, and diarrhea infection in children can quickly progress to severe disease and death.^8^ The prompt administration of an efficacious antimalarial or antibiotic for pneumonia and oral rehydration salts and zinc (ORS/zinc) for diarrhea as required, is therefore essential to prevent death or lasting sequelae. Although uncomplicated malaria or pneumonia infection can be easily treated with oral medication, severe disease requires parenteral treatment alongside the management of complications, which is usually only available in a higher-level health facility setting.^9^ Children who develop severe malaria or pneumonia in remote settings are therefore at risk of death, if symptoms are not recognized and treated early.

Community-level initiatives such as the integrated community case management (iCCM) of malaria, pneumonia, and diarrhea or the Integrated Management of Childhood Illnesses (IMCI) in primary health facilities therefore seek to facilitate prompt identification of children with signs of severe disease and immediate referral to a health facility with the capacity to manage the disease and its complications.^10^^,^^11^ The danger signs of severe disease in children < 5 years of age that should trigger prereferral treatment and immediate referral within iCCM programs include a child who is unconscious/unusually sleepy, unable to feed or drink, vomits everything, and one who has convulsions.^3^

Treatment-seeking practices for children with febrile illness have been extensively described, but less is understood about the care-seeking patterns and treatment provided to those children with danger signs of severe disease, including severe malaria and pneumonia.^12^ Understanding treatment-seeking patterns in the case of severe illness is important to target relevant interventions adequately, including prereferral treatment, such as rectal artesunate for cases of severe malaria in children. The objective of this study was therefore to determine the treatment seeking practices and treatment patterns for children < 5 years of age with an acute febrile illness, with and without danger signs of severe disease, in a highly malaria-endemic area of northern Uganda.

## METHODS

In the frame of the Community Access to Rectal Artesunate for Malaria (CARAMAL) Project, an observational study accompanying the rollout of rectal artesunate as prereferral treatment of severe malaria, cross-sectional household surveys were conducted to elicit treatment seeking for recently sick children and their treatment patterns.^13^ The cross-sectional surveys were designed to collect contextual information relevant for the CARAMAL study.

### Study design.

Three consecutive household surveys were conducted between November and December each year, in 2018, 2019, and 2020 in northern Uganda.

### Study setting.

The surveys were conducted in the districts of Kole, Kwania, and Oyam, located in the Lango Region of northern Uganda. The three districts together have a population of approximately one million, and more than 80% of the population are rural subsistence farmers.^14^ Malaria is endemic, with all year transmission and a high entomological inoculation rate of more than 1,500 infective bites per person per year.^15^ Health services are provided by public health facilities including 30 primary health facilities (health center II), 15 secondary health facilities (health center III), and four tertiary health facilities (health center IV and referral hospitals), as well as approximately 5,000 community health workers (implementing iCCM), and many small, private, for-profit facilities. Treatment of uncomplicated conditions including malaria, diarrhea, and pneumonia are provided at all levels including the CHWs, whereas health center facilities of level III and higher provide inpatient services. Since March 2019, CHWs and primary health centers (HC II) have been supplied with rectal artesunate for prereferral treatment of children with suspected severe malaria.^13^

Uganda’s climate is largely tropical with two rainy seasons per year, March to May and September to December. Data collection took place from the middle to the end of the rainy season when prevalence of malaria is expected to be high.

### Sampling and sample size.

A stratified three-stage cluster sampling strategy was applied to randomly sample survey households (Table 1). Stratification was by district, and the sampling stages included the parish, village, and household. A total of 34 parishes/clusters were sampled from the three districts, with the number proportional to the population size of the district. One village was then sampled from each cluster and within each village, 30 households were randomly sampled from a list of households with children < 5 years of age established by the survey team and village representatives before commencing data collection. At each village, we first mapped and listed all households 1 to 3 weeks before the actual survey. Information on the household location, household head, number of people in the household, and number of children under age 5 was obtained. The “sampling frame” included all households with at least one child below 5 years, from which we randomly sampled 30 households per village. When there was more than one eligible child per caregiver, one was randomly selected from the list of eligible children. This was predesigned and automatically generated using data collection software.

**Table 1:** Household survey sampling, based on 2014 census population

District	Total population	No. of parishes	No. of villages	No. of households	Survey sample/no. of parishes (total samples = 34)
Oyam	383,644	64	1038	76,536	16
Kwanya	183,930	34	396	34,400	9
Kole	239,327	30	436	48,525	9

To determine the proportion of children aged < 5 years seeking treatment for fever from formal health facilities, we used an estimated prevalence of treatment seeking of 50% with 95% confidence, α = 0.05 and a design effect of 1.2, giving a minimum of 462 household survey responses on treatment-seeking required per individual survey round.

The number of clusters was determined using the WHO expanded program of immunization recommendation of 30 cluster * 30 interviews per cluster. We used 34 clusters to allow sample size achievement for additional objectives.

### Study population.

Parents/caregivers of children < 5 years of age were eligible to participate in face-to-face interviews if their child had been ill with a febrile illness within 2 weeks before the survey. Additional household-level information was obtained in interviews with the heads of sampled households (who may also have been the parent/caregiver).

### Data collection.

Trained field interviewer teams collected information in electronic data collection forms using the Open Data Kit (ODK) software on password protected tablets with customized data entry screens. Data collection forms were adapted from malaria indicator survey templates. Interviews were conducted in English or the local Langi, depending on the preference of the respondent. Electronic forms in either language were available. The forms collected the child’s demographic information and data on the child’s symptoms including danger signs. Danger signs were defined following the WHO IMCI/iCCM guidelines: unconscious or unusually sleepy, unable to feed or drink, vomiting everything, and convulsions.^11^ Because of the lack of specificity and sensitivity of questions related to the severity and frequency of vomiting, “vomiting everything” may have been overreported.

Data collectors also collected data on the type and location of healthcare providers visited and the type of medicine received. Charts with photographs of the common medicines used to treat febrile illnesses (including ACT and antibiotics) were shown to the respondents to aid their memory of the medicines that the child was given for treatment.

### Analysis.

Only children who were reported to have been sick with a febrile illness within 2 weeks before the household survey were included in this analysis. All analyses took into consideration the cluster sampling strategy through weighting, using the “svyset” command (with village of residence as the cluster) in Stata, version 14. Descriptive analyses of point estimates of indicators were conducted. Most variables are presented as proportions, disaggregated by survey year and severity of illness (danger sign versus no danger sign). Finally, multivariate logistics regression analysis was conducted to adjust for possible confounders and to determine differences between children with and without danger signs. The following variables were included in the multivariable logistics regression model: district of residence, household survey round, child age, child gender, and caregiver age and caregiver gender.

### Ethics.

Ethical approval was obtained from Makerere University School of Public Health Higher Degrees Research and Ethics Committee (Number 548); from the Uganda National Council of Science and Technology (SS 4534) and from the Research Ethics Review Committee at the World Health Organization in Geneva (ERC.0003008). Written informed consent was obtained from all participants including the head of the household and the child caregiver before data collection.

## RESULTS

### Characteristics of the study population.

The number of children < 5 years reported to have had a febrile illness within the past 2 weeks amounted to 398 in 2018, 416 in 2019, and 547 in 2020. The mean age of these children was 2.5 years (SD = 1.4), and 52% were female. All the children had a fever, 46% had cough, and 19% had diarrhea during the reported illness episode (Table 2).

**Table 2:** Characteristics of children with any reported illness, 2 weeks before the survey

	Survey			
	2018	2019	2020	Overall
Number of recently febrile children	398	416	547	1,361
Age (Mean, SD)	2.3 (1.4)	2.7 (1.3)	2.4 (1.4)	2.5 (1.4)
Sex (Female %)	54.6	51.7	50.3	52.2
District (%)				
Kole	23.1	25.0	19.9	22.6
Oyam	51.5	48.8	54.8	51.7
Kwania	25.4	26.2	25.2	25.6
Major signs and symptoms (%)				
Cough	51.3	49.3	38.7	46.4
Fast breathing	2.5	3.6	1.5	2.5
Diarrhea	21.6	16.7	17.8	18.7
Danger signs (%)*	27.4	32.0	31.4	30.3
Convulsions	2.3	2.3	0.4	1.7
Unconscious/unusually sleepy	6.6	4.4	9.9	6.3
Unable to feed or drink	10.4	7.8	5.0	7.7
Vomiting everything	12.9	18.7	18.2	16.6

* Prevalence of danger signs (any danger sign/total number of children). A child could have more than one danger sign. Therefore, total of the individual danger signs may be higher than the prevalence.

Overall, 30% were reported to have had at least one danger sign of severe disease. These included about 2% with convulsions, 6% who were unconscious/unusually sleepy, 8% who were unable to feed or drink, and 17% who had been vomiting everything. See Table 2 for more details of the children in the study.

### Care-seeking patterns for children with and without WHO-classified danger signs.

Overall, a high number of children were treated at home with medicines obtained/kept at home. These were 53% over the three survey rounds, combined (Table 3). There was no difference in the proportion of children with and without danger signs who received treatment at home (adjusted odds ratio [aOR]: 0.83; 95% confidence interval [CI]: 0.66–1.05; *P* = 0.13). However, significantly more children with danger signs sought treatment outside the home compared with those without danger signs (aOR: 1.6; 95% CI: 1.2–2.0; *P* < 0.001) (Table 3).

**Table 3:** Care seeking and treatment of children with and without danger signs

	No danger sign	Danger sign	Total	Adjusted odds ratio (95% CI)*	*P* value
	*n* = 945	*n* = 416	*n* = 1,361		
Did something at home	54.2	49.3	52.7	0.83 (0.66–1.05)	0.13
Took medicine	30.8	25.2	29.1	0.73 (0.55–0.95)	0.03
Took antimalarial	23.2	21.4	22.6	0.87 (0.65–1.15)	0.33
Artemether–lumefantrine	22.5	21.4	22.2	0.9 (0.68–1.2)	0.49
Sought treatment outside home	50.6	61.8	54.0	**1.6 (1.2–2.0)**	**< 0.001**
Type of outside provider visited first†					
Community health worker	11.2	13.0	11.8	1.2 (0.8–1.7)	0.41
Referral facility	1.9	1.9	1.9	0.8 (0.3–1.8)	0.55
Secondary/primary facility	10.2	16.6	12.1	**1.7 (1.2–2.4)**	**0.002**
Clinic	16.6	20.0	17.6	1.3 (1.0–1.8)	0.06
Drug shop	6.2	5.3	6.0	0.9 (0.5–1.4)	0.58
Blood test done outside home	37.0	48.3	40.5	**1.6 (1.2–2.0)**	**< 0.001**
Received antimalarial outside home	34.7	48.6	38.9	**1.8 (1.4–2.2)**	**< 0.001**
Artemether–lumefantrine	31.3	41.6	34.5	**1.5 (1.2–2.0)**	**0.001**
Rectal artesunate	0.1	0	0.1		
Received antibiotic outside home	9.6	9.4	9.6	0.97 (0.7–1.4)	0.887

CI = confidence interval.

* Adjusted for survey round, district of residence, child age, child sex, and caretaker age, and caretaker sex. The bold text represents statistically significant results.

† A small percentage also sought care at “other” sources, including from friends, traditional healers, and places of worship.

The first source of care for children who sought care outside the home is also presented in Table 3. Overall care seeking from CHWs was low. This was on average only 12% (12% in 2018, 6% in 2019, and 14% in 2020). Care seeking at the referral health facility was also only 2% (1%, 1%, and 2% over the three survey rounds, respectively). Further, care seeking from a primary or secondary facility was 12% of the total children surveyed. That is 7%, 12%, and 13%, respectively, across the three survey rounds (Table 3). In total, across the three survey rounds, care seeking from the public sector (including the CHW and the health facilities) was 26% of all the sick children.

Meanwhile, care seeking in the private sector included both private sector clinics and drug stores. A total of 18% of the sick children sought care at a private clinic overall. These were 16%, 21%, and 21% in 2018, 2019, and 2020 respectively. Those seeking care at drug stores were 6% in total (6%, 8%, and 4% over the three survey rounds, respectively). In total across the survey rounds, seeking care from private sector providers amounted to 24%. Overall, a similar number of children sought care in the public sector (26%) as in the private sector (24%).

### Treatment patterns.

The use of diagnostic tests/blood tests was also higher among children with a WHO-classified danger sign (48%), compared with those without danger signs (37%) across all three survey rounds (Table 3). Children with danger signs had 60% higher odds of receiving a diagnostic test (aOR: 1.6; 95% CI: 1.2–2.0; *P* < 0.001). The malaria test was positive in 37% of the children across the survey rounds, and more children with danger signs had a positive malaria test (47%) than those without danger signs (33%; *P* < 0.001).

Those who were reported to have received antimalarial medicines outside the home were 39% across the survey rounds. Again, more children with danger signs received the antimalarial treatment compared with those without danger signs (aOR: 1.8; 95% CI: 1.4–2.2; *P* < 0.001). The use of rectal artesunate was low, with less than 1% of the children receiving the suppository over all the survey rounds.

Reported antibiotic use (from an outside health provider) was generally low, 10%, and similar across the two groups (danger sign/no danger sign) over the 3 years (aOR: 0.97; 95% CI: 0.7–1.4; *P* = 0.89). See Table 3 for additional information on care-seeking and treatment patterns.

Finally, treatment of diarrhea with ORS/zinc was very low throughout the study period, and only about 5% of the children with diarrhea received this medication.

## DISCUSSION

This study looked at treatment-seeking patterns and treatment provided to children with and without danger signs of severe disease, to identify patterns relevant for improving management of children in rural communities in Uganda. Data from three household surveys conducted in 2018, 2019, and 2020 were used to derive and compare care-seeking and treatment patterns. The big data sets, multiple rounds, and generally consistent results across the survey rounds contributed to the strength of the results and information presented in this paper.

Thirty percent (30.3%) of the children in this study were reported to have danger signs of severe disease. This figure is slightly higher than what has been previously reported in other studies that actually assess and report severity of symptoms in children < 5 years of age who visit CHWs, in similar settings. These studies in Burkina Faso, Nigeria, and Uganda have reported danger signs in 18% to 25% of the children.^16^ The higher number with danger signs in this study could be due to overinterpretation of a child’s symptoms by a caregiver as more severe than they actually are—for example, general weakness, sleepiness, and vomiting that could have been reported as vomiting all feeds. In addition, due to a lack of specificity and sensitivity of questions related to severity and frequency of vomiting, nonsevere vomiting may have been reported as “vomiting everything.”

Despite a high prevalence of reported danger signs, only half of all children (including those with danger signs) actually received care from a health provider outside their home. In addition, half of all the children were treated with medicines or remedies found at home. This is similar to findings from a treatment-seeking review that included 19 studies from Uganda that found home management of children to be a common first response in management of sick children.^17^ Although it is expected that caretakers should provide first aid to a sick child at home, it is possible that many of the children may not receive appropriate treatment at their homes and may also have delayed care seeking after initial remedies at home.

Other studies on treatment seeking highlight common factors that influence mothers’ healthcare-seeking behavior for their children. These include social and economic factors, particularly higher maternal education and employment status of mothers contribute to better care seeking for children.^18^^,^^19^ On the contrary, low economic status leading to lack of money to travel and sustain patient admission, as well as lack of immediate transport, directly contribute to poor care-seeking behavior. Other child factors such as child’s age are associated with care-seeking behavior.^20^ Many of these factors, especially the socioeconomic factors, are also prevalent in our study setting and may explain why care was not sought for many children in this study immediately after symptoms were recognized. There is therefore a need to address socioeconomic barriers to access to care and to increase caretaker awareness on the urgency of recognition and prompt action when danger signs of disease are present in children.

This study also found equal care seeking in the public sector (26%) and the private sector (24%). Many interventions are targeted at improving care provision in the public sector, the private sector is often left behind and no training, quality improvement opportunities, or support supervision is provided to health workers in the private sector.^21^ Given that the private sector treats as many patients as the public sector, it is necessary to ensure that training is provided to the health workers and quality medicines and diagnostics are made available through the private sector in rural communities in Uganda and similar countries.^22^

Further, the fairly low utilization of community health workers (12%) found in this study could be improved. Given that CHWs are often nearer to families than health facilities, ensuring that the CHWs are well trained and supplied with the necessary medicines, diagnostics, and equipment can contribute to timely access to appropriate treatment of children with fever in their communities.^23^^–^^25^

On another note, this study found low reported use of antibiotics in febrile children (10%). In this setting with very high malaria transmission, febrile illness may generally be first treated as malaria. On the contrary, many studies show high prescription and use of both antibiotics and antimalarial medicines to treat febrile illness in children.^26^^,^^27^

Finally, an additional limitation of this work should be highlighted. As is common in surveys on treatment seeking, these data are based on caregiver-reported information that could not be verified by the study team. However, to improve the data quality, the period of recall was limited to 2 weeks before the survey. In addition, pictures of common medicines used for treatment of children were shown to respondents to help improve their memory of the medicines that the children may have been taking.

## CONCLUSION

In conclusion, this study found high prevalence of severe disease in children and yet low care seeking in general. Understanding and addressing barriers to access to health care could contribute to better treatment seeking practices.

## References

[1] Lozano R , 2012. Global and regional mortality from 235 causes of death for 20 age groups in 1990 and 2010: a systematic analysis for the Global Burden of Disease Study 2010. Lancet 380: 2095–2128. 23245604 10.1016/S0140-6736(12)61728-0 PMC10790329

[2] United Nations , 2015. The UN InterAgency Group for Child Mortality Estimation—Levels and Trends in Child Mortality. Report 2015. New York, NY: United Nations Children’s Fund.

[3] MoH , 2010. Integrated Community Case Management of Childhood Malaria, Pneumonia and Diarrhoea: Implementation Guide. Kampala, Uganda: Ministry of Health CHD, ed.

[4] MoH , 2020. Ministry of Health Annual Health Sector Performance Report 2019/2020. Kampala, Uganda: Ministry of Health, Uganda.

[5] Kallander K Nsungwa-Sabiiti J Peterson S, 2004. Symptom overlap for malaria and pneumonia—policy implications for home management strategies. Acta Trop 90: 211–214. 15177148 10.1016/j.actatropica.2003.11.013

[6] WHO, UNICEF , 2004. Management of Pneumonia in Community Settings. Geneva, Switzerland: World Health Organization/United Nations Children’s Fund.

[7] WHO , 2020. World Malaria Report. Geneva, Switzerland: World Health Organization.

[8] Mackintosh CL Beeson JG Marsh K, 2004. Clinical features and pathogenesis of severe malaria. Trends Parasitol 20: 597–603. 15522670 10.1016/j.pt.2004.09.006

[9] WHO , 2015. Guidelines for the Treatment of Malaria. Geneva, Switzerland: World Health Organization.

[10] Young M Wolfheim C Marsh DR Hammamy D, 2012. World Health Organization/United Nations Children’s Fund Joint Statement on integrated community case management: an equity-focused strategy to improve access to essential treatment services for children. Am J Trop Med Hyg 87: 6–10. 23136272 10.4269/ajtmh.2012.12-0221 PMC3748523

[11] WHO , 2005. Integrated Management of Childhood Illness—Handbook. Geneva, Switzerland: World Health Organization.

[12] Brunner NC Awor P Hetzel MW, 2021. Definitions of severity in treatment seeking studies of febrile illness in children in low and middle income countries: a scoping review. Int J Public Health 66: 634000. 34526874 10.3389/ijph.2021.634000 PMC8435535

[13] Lengeler CBC , 2021. Community access to rectal artesunate for malaria (CARAMAL): a large-scale observational implementation study in the Democratic Republic of the Congo, Nigeria and Uganda. medRxiv 2021.12.10.21266567. 10.1371/journal.pgph.0000464 PMC10022208 36962706

[14] UBOS , 2018. Uganda Demographic Health Survey 2016. Kampala, Uganda, and Rockville, MD: Uganda Bureau of Statistics (UBOS) and ICF.

[15] Okello PE Van Bortel W Byaruhanga AM Correwyn A Roelants P Talisuna A D’Alessandro U Coosemans M, 2006. Variation in malaria transmission intensity in seven sites throughout Uganda. Am J Trop Med Hyg 75: 219–225. 16896122

[16] Ajayi IO , 2016. Feasibility of malaria diagnosis and management in Burkina Faso, Nigeria, and Uganda: a community-based observational study. Clin Infect Dis 63: S245–S255. 27941101 10.1093/cid/ciw622 PMC5146694

[17] Kassam R Collins JB Liow E Rasool N, 2015. Caregivers’ treatment-seeking behaviors and practices in Uganda—a systematic review (Part II). Acta Trop 152: 269–281. 26259818 10.1016/j.actatropica.2015.07.029

[18] Adedokun ST Yaya S, 2020. Factors influencing mothers’ health care seeking behaviour for their children: evidence from 31 countries in sub-Saharan Africa. BMC Health Serv Res 20: 842. 32894107 10.1186/s12913-020-05683-8 PMC7487813

[19] Akinyemi JO Banda P De Wet N Akosile AE Odimegwu CO, 2019. Household relationships and healthcare seeking behaviour for common childhood illnesses in sub-Saharan Africa: a cross-national mixed effects analysis. BMC Health Serv Res 19: 308. 31088474 10.1186/s12913-019-4142-x PMC6518738

[20] Price J Lee J Willcox M Harnden A, 2019. Place of death, care-seeking and care pathway progression in the final illnesses of children under five years of age in sub-Saharan Africa: a systematic review. J Glob Health 9: 020422. 31673338 10.7189/jogh.09.020422 PMC6815655

[21] Awor P Wamani H Bwire G Jagoe G Peterson S, 2012. Private sector drug shops in integrated community case management of malaria, pneumonia, and diarrhea in children in Uganda. Am J Trop Med Hyg 87: 92–96. 23136283 10.4269/ajtmh.2012.11-0791 PMC3748528

[22] Awor P Peterson S Gautham M, 2018. Delivering child health interventions through the private sector in low and middle income countries: challenges, opportunities, and potential next steps. BMJ 362: k2950. 30061154 10.1136/bmj.k2950 PMC6063308

[23] Young M Sharkey A Aboubaker S Kasungami D Swedberg E Ross K, 2014. The way forward for integrated community case management programmes: a summary of lessons learned to date and future priorities. J Glob Health 4: 020303. 25520789 10.7189/jogh.04.020303 PMC4267099

[24] Rasanathan K Bakshi S Rodriguez DC Oliphant NP Jacobs T Brandes N Young M, 2014. Where to from here? Policy and financing of integrated community case management (iCCM) of childhood illness in sub-Saharan Africa. J Glob Health 4: 020304. 25520790 10.7189/jogh.04.020304 PMC4267097

[25] Seidenberg PD Hamer DH Iyer H Pilingana P Siazeele K Hamainza B MacLeod WB Yeboah-Antwi K, 2012. Impact of integrated community case management on health-seeking behavior in rural Zambia. Am J Trop Med Hyg 87: 105–110. 23136285 10.4269/ajtmh.2012.11-0799 PMC3748509

[26] Hopkins H , 2017. Impact of introduction of rapid diagnostic tests for malaria on antibiotic prescribing: analysis of observational and randomised studies in public and private healthcare settings. BMJ 356: j1054. 28356302 10.1136/bmj.j1054 PMC5370398

[27] Elven J , 2020. Non-malarial febrile illness: a systematic review of published aetiological studies and case reports from Africa, 1980–2015. BMC Med 18: 279. 32951596 10.1186/s12916-020-01744-1 PMC7504660

